# Recurrent *MBTPS2* variant c.970+5G>A in IFAP syndrome: a mutational hotspot

**DOI:** 10.1038/s41439-026-00346-2

**Published:** 2026-04-14

**Authors:** Sheetal Kumar, Sohail Ahmed, Pietro Incardona, Nicole Cesarato, Yue Zhang, Monica Ines Natale, Muhammad Javed Khan, Laura Valinotto, Kifayat Ullah, Wasim Ahmad, Ines Irurzun, Peter M. Krawitz, Bo Liang, Regina C. Betz

**Affiliations:** 1 Institute of Human Genetics, Medical Faculty, University Hospital Bonn, University of Bonn, Bonn, Germany; 2 Institute of Biochemistry, University of Balochistan, Quetta, Pakistan; 3 Institute for Genomic Statistics and Bioinformatics, Medical Faculty, University Hospital Bonn, University of Bonn, Bonn, Germany; 4 Department of Clinical Laboratory, The First Affiliated Hospital, Anhui Medical University, Hefei, China; 5 Center for Research in Genodermatosis and Epidermolysis Bullosa, School of Medicine, University of Buenos Aires, Buenos Aires, Argentina; 6 Consejo nacional de investigaciones científicas y tecnicas, Buenos Aires, Argentina; 7 Department of Biochemistry, Faculty of Biological Sciences, Quaid-i-Azam University, Islamabad, Pakistan; 8 Unidad de Dermatología, Hospital de Niños Dr. Ricardo Gutiérrez, Buenos Aires, Argentina; 9 Department of Dermatology, The First Affiliated Hospital of Anhui Medical University, Hefei, China

**Keywords:** Genetic markers, Diseases, Haplotypes

## Abstract

Ichthyosis follicularis, alopecia and photophobia (IFAP) syndrome type I is a rare, X-linked disorder resulting from pathogenic variants in *MBTPS2*. Here we report a Pakistani IFAP pedigree of three affected individuals harboring the recurrent *MBTPS2* splice-site variant c.970+5G>A that was reported previously in Chinese and Argentinian families. Haplotype analyses across these three families excluded a founder effect, establishing c.970+5G>A as a recurrent mutational hotspot. In addition, phenotypic severity varied across the families, suggesting additional modifiers.

## Data report

Ichthyosis follicularis, alopecia and photophobia (IFAP) syndrome type I is a rare, X-linked genodermatosis characterized by follicular hyperkeratosis, congenital nonscarring alopecia and photophobia. IFAP is often accompanied by palmoplantar keratoderma, nail dystrophy and variable systemic features. These may include short stature, intellectual disability, seizures, recurrent infections and dysmorphic facial features such as frontal bossing, large ears or choanal atresia. Gastrointestinal anomalies such as Hirschsprung’s disease or omphalocele, as well as skeletal abnormalities and renal malformations, have also been reported. In its most severe form, IFAP syndrome may present as BRESHECK syndrome (brain anomalies, retardation, ectodermal dysplasia, skeletal malformations, Hirschsprung disease, ear-eye anomalies, cleft palate/cryptorchidism and kidney dysplasia). The disorder results from pathogenic variants in the gene *MBTPS2*, which is located on chromosome Xp22.12-p22.11. *MBTPS2* encodes the membrane-bound transcription factor site-2 protease, a zinc metalloprotease critical for cholesterol homeostasis and the endoplasmic reticulum (ER) stress response. Disruption of MBTPS2 function impairs the regulated intramembrane proteolysis of sterol regulatory element-binding proteins (SREBPs). This results in defective lipid metabolism and cellular stress adaptation, particularly in tissues such as skin and hair follicles^1^.

**Fig. 1:**
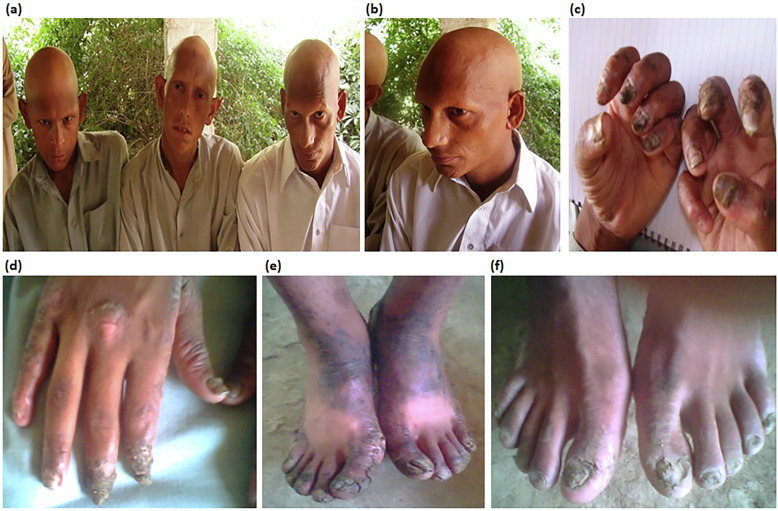
Clinical features of IFAP syndrome in the Pakistani family. **a**, **b** Universal nonscarring alopecia affecting the scalp, eyebrows and eyelashes in all individuals. **c**–**f** Hands and feet of the affected brothers, showing palmoplantar ungual hyperkeratosis, nail dystrophy and dyschromia.

**Fig. 2:**
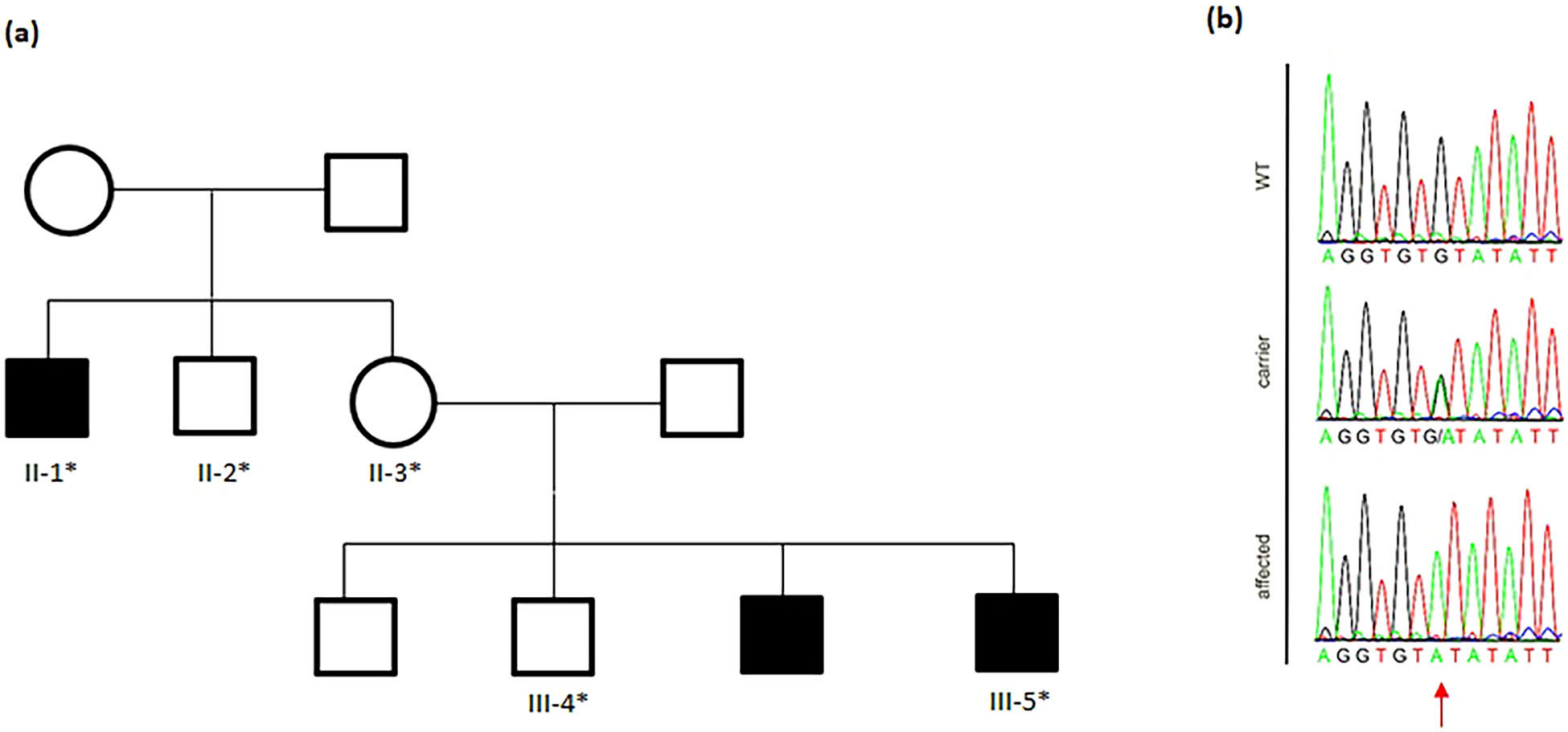
Pedigree and molecular genetic findings in the Pakistani IFAP family. **a** Pedigree of the family, illustrating X-linked inheritance of IFAP syndrome type I. Individuals who underwent sequencing are marked with an asterisk. **b** Sanger sequencing chromatograms depicting the *MBTPS2* c.970+5G>A variant (red arrow) in wild-type (WT), heterozygous carrier, and affected (hemizygous) individuals.

The present report describes a Pakistani family with IFAP syndrome. The affected individuals comprised two brothers and their maternal uncle. All three affected individuals exhibited the classic manifestations of IFAP: widespread follicular hyperkeratosis; universal alopecia involving the scalp, eyebrows and eyelashes; and early-onset photophobia (Figs. 1a, b and 2a). Additional features included palmoplantar ungual hyperkeratosis with dystrophy and dyschromia (Fig. 1c–f). No neurodevelopmental anomalies or other features associated with the severe BRESHECK syndrome were present. The unaffected mother was an asymptomatic carrier with no cutaneous or ocular abnormalities, consistent with X-linked inheritance.

Molecular genetic analysis via Sanger sequencing identified the hemizygous *MBTPS2* (NM_015884.4) splice-site variant c.970+5G>A in the index patient and the affected maternal uncle (Fig. 2b). According to American College of Medical Genetics and Genomics variant classification guidelines, this variant is classified as likely pathogenic (PVS1_strong, PP1, PM2_supporting). The variant occurs outside the canonical ±1–2 splice site. However, loss of function is a known disease mechanism for *MBTPS2*-related IFAP, and previous cDNA analysis demonstrated that this variant disrupts the canonical donor site of exon 7, resulting in partial exon skipping (r.951_970del), with a predicted frameshift and premature stop codon (p.Gln317Hisfs*9)^2^. For the second affected brother, no material was available for genetic testing. However, familial segregation analysis confirmed the mother as a heterozygous carrier, and excluded the variant in two unaffected male relatives (another maternal uncle and a further brother of the index patient).

**Fig. 3:**
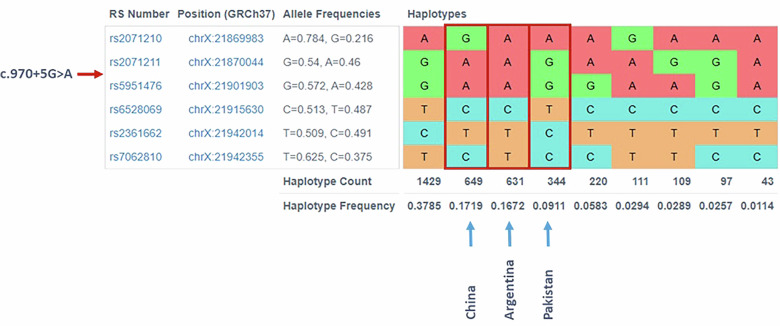
Haplotype analysis at the *MBTPS2* locus in families with the c.970+5G>A variant. LDhap Tool screenshot of the *MBTPS2* region (red arrow) spanning approximately ±0.2 Mb around the gene in the three unrelated families from Pakistan, Argentina and China. Each column represents a haplotype observed in population reference controls and each row a single nucleotide variant (SNV); different colors indicate different alleles at each SNV position. Distinct haplotypes are observed for each family, demonstrating the absence of a shared founder haplotype and supporting independent mutational events (blue arrows).

The c.970+5G>A variant has now been independently identified in three ethnically distinct families from Pakistan, China and Argentina^2,3^. To investigate whether the recurrent appearance of c.970+5G>A in these families reflects a mutational hotspot or a founder effect, written informed consent was obtained from all participants (Pakistani, Argentinian and Chinese probands), in accordance with the Declaration of Helsinki. Genome-wide genotyping was performed using GSAMD24v3-0 chips (Ilumina), and the Infinium HTS Assay protocol (Ilumina). Phasing of the genotypes was conducted using Eagle 2.4.1 and the 1000 Genomes Phase 3 dataset as a reference panel^4,5^. Haplotypes were reconstructed in a 0.2 megabase (Mb) region around *MBTPS2* on chromosome X. Using the LDhap Tool, three completely distinct haplotype architectures were identified (Fig. 3). This excludes a shared ancestral origin, and supports the hypothesis of recurrent, independent mutational events.

The independent occurrence of c.970+5G>A across unrelated families and haplotype analyses exclude a founder effect, and strongly suggest that this site represents a true mutational hotspot, that is, a site that is particularly prone to acquiring new pathogenic variants. Several mechanisms can contribute to the formation of these hotspots. These include intrinsic DNA sequence properties (for example, CpG dinucleotides prone to deamination); replication slippage at repetitive sequences; local secondary structures (for example, G-quadruplexes, and hairpins); and DNA repair inefficiencies^6,7^. Furthermore, genomic regions located in late-replicating domains are subject to an increased mutational burden^6^. Although c.970+5G>A does not reside within a CpG dinucleotide (thus ruling out methylation-mediated deamination), the variant represents a G > A transition on the coding strand. While reactive oxygen species can induce a variety of DNA lesions affecting multiple bases, guanine (G) is particularly susceptible to oxidative damage, even outside CpG contexts, due to its low redox potential^8^. In addition, the local sequence context surrounding c.970+5G>A is part of a GTGTGT motif, which may promote polymerase stalling during replication, a mechanism known to increase replication errors at repetitive sequences^9^.

While all carriers of the c.970+5G>A variant exhibited severe oculocutaneous involvement, phenotypic variability was noted across the three families. While the Argentinian patient exhibited neurodevelopmental anomalies and absence seizures, the Chinese and Pakistani patients did not. Our group and others have previously described a similar observation for another *MBTPS2* variant, whereby German and Japanese patients harboring c.1286G>A exhibited marked variability in disease severity^10^. Interindividual variability among families harboring the same pathogenic variant strongly suggests the influence of genetic or environmental modifiers. Biologically, potential genetic modifiers may involve components of *MBTPS2*-dependent signaling cascades, particularly the *SREBP* pathway and regulators of ER stress^1^. Notably, comparable phenotypic variability has been reported in autosomal IFAP cases with variants in *SREBP1*^11^. Environmental and epigenetic influences that modulate lipid homeostasis and ER stress, such as infections, systemic inflammation, oxidative stress or metabolic status are likely to further modify phenotype severity. In female carriers, an additional layer of variability is added by X-inactivation. Skewed inactivation can determine whether heterozygotes remain clinically unaffected or develop mosaic or even more generalized cutaneous manifestations^12^. No genotype–phenotype correlation can be inferred from splice‑site versus missense variants alone, as both variant types have been associated with comparable clinical spectra of variable severity. Previous studies have highlighted the importance of conserved motifs within the MBTPS2 protein, such as the HEIGH and LDG motifs, which are critical for coordinating the zinc atom at the enzyme’s active site^13^. Genotype–phenotype correlations suggest that variants located near hydrophobic, intramembranous domains encompassing the LDG motif are typically associated with more severe IFAP phenotypes^13,14^. Similarly, variants within specific transmembrane domains, such as p.Trp226Leu and p.His227Leu in TM5, have been associated with a severe form of IFAP^9^. Nevertheless, the present findings, in conjunction with those from previous reports, demonstrate that substantial phenotypic heterogeneity can occur even among patients with identical *MBTPS2* variants, as illustrated by both c.970+5G>A and c.1286G>A. This underscores the hypothesis that additional genetic, epigenetic or environmental factors shape the clinical expression of *MBTPS2*-related disorders.

In conclusion, the present findings establish c.970+5G>A as a recurrent mutational hotspot. The absence of a founder effect, and the presence of distinct haplotypes, suggest that sequence-specific vulnerabilities are the primary driver of the recurrent variant. Future studies are required to identify genetic, epigenetic and environmental modifiers of phenotypic expression.

## HGV Database

The relevant data from this Data Report are hosted at the Human Genome Variation Database at 10.6084/m9.figshare.hgv.3637.
